# Biodegradation of L-Valine Alkyl Ester Ibuprofenates by Bacterial Cultures

**DOI:** 10.3390/ma14123180

**Published:** 2021-06-09

**Authors:** Edyta Makuch, Paula Ossowicz-Rupniewska, Joanna Klebeko, Ewa Janus

**Affiliations:** Department of Chemical Organic Technology and Polymeric Materials, Faculty of Chemical Technology and Engineering, West Pomeranian University of Technology, Szczecin, PL-70322 Szczecin, Poland; emakuch@zut.edu.pl (E.M.); joanna.klebeko@gmail.com (J.K.); ejanus@zut.edu.pl (E.J.)

**Keywords:** ionic liquids, biodegradability, biotechnology, ibuprofen, L-valine alkyl ester ibuprofenates, *log* P, non-steroidal anti-inflammatory drug

## Abstract

Nowadays, we consume very large amounts of medicinal substances. Medicines are used to cure, halt, or prevent disease, ease symptoms, or help in the diagnosis of illnesses. Some medications are used to treat pain. Ibuprofen is one of the most popular drugs in the world (it ranks third). This drug enters our water system through human pharmaceutical use. In this article, we describe and compare the biodegradation of ibuprofen and ibuprofen derivatives—salts of L-valine alkyl esters. Biodegradation studies of ibuprofen and its derivatives have been carried out with activated sludge. The structure modifications we received were aimed at increasing the biodegradation of the drug used. The influence of the alkyl chain length of the ester used in the biodegradation of the compound was also verified. The biodegradation results correlated with the lipophilic properties (*log* P).

## 1. Introduction

Due to their high effectiveness in pain management strategies, non-steroidal anti-inflammatory drugs are a widely used group of drugs in the world. Ibuprofen went to treatment over 50 years ago and has become one of the most popular non-steroidal anti-inflammatory drugs in the world. This compound is characterized by high lipophilicity. In 2000, ibuprofen consumption reached 300 tons in Germany, 162 tons in England, 58 tons in Poland, and 25 tons in Switzerland [1]. It is worth noting that ibuprofen consumption constantly increases. The global ibuprofen market was valued at US$294.4 million in 2020, and is expected to reach US$447.6 million by the end of 2026. As a result of increased production, consumption, and easy availability of NSAIDs, the pollution of the environment with these substances increases, which often end up in wastewater and then into waters in an unchanged composition [2,3,4]. The release of these drugs into the environment poses a real threat to animals, humans, and the ecosystem [5,6,7].

Few literature reports have described the degradation of nonsteroidal anti-inflammatory drugs (including ibuprofen) using the active sludge. The most popular method of degradation is based on the decomposition of ibuprofen by ligninolytic species of mushrooms [8]. However, hydroxylated derivatives of ibuprofen (1-hydroxyibuprofen, 2-hydroxyibuprofen, 1,2-dihydroxyibuprofen) formed as a result of degradation may have higher toxicity than the parent compound [9]. Nonetheless, in the case of the biotransformation of ibuprofen (by the Nocardia sp. NRRL 5646 strain), metabolites of non-steroidal anti-inflammatory drugs were ibuprophenyl and ibuprophenyl acetate, which were further mineralized [10]. An interesting solution that eliminates the formation of these derivatives of ibuprofen is the biodegradation of ibuprofen utilizing aerobic microorganisms (*Sphingomonas strain*) that treat the test compound as the only source of carbon and energy [11]. In the first stage, thioesterification of ibuprofen takes place in the presence of CoA ligase, resulting in ibuprophenyl-CoA. Then, as a result of the deoxygenation of the aromatic ring, 4-isobutyl catechol is formed. In the next stage of research, the catechol system was split, resulting in 2-hydroxy-5-isobutylhexa-2,4-diene acid [11,12].

Low solubility and bioavailability are factors that limit the effectiveness of many drugs. Various structural drug modifications are used to change these parameters. For example, antiviral nucleoside analogues derived from valine esters such as valine ester prodrugs of acyclovir, ganciclovir, cyclopropavir, and valganciclovir are known [13,14,15,16,17,18,19]. Moreover, it is well known that D-valine tert-butyl ester hydrochloride is used as an intermediate in pharmaceuticals and as an antitumor drug. L-valine alkyl ester hydrochlorides are also used in the synthesis of peptides [20,21]. However, no research has been undertaken on the biodegradation of these compounds.

In our previous publications, we described the synthesis, properties, skin penetration, and skin accumulation of new ibuprofen derivatives—L-valine alkyl ester ibuprofenates [22,23]. It has been shown that these compounds have a much higher solubility in water and body fluids and better skin permeability, therefore being an alternative to ibuprofen. Due to numerous advantages, it was decided to verify how these compounds can affect the environment, in order to investigate the effect of changing the form of a compound from acidic-ibuprofen to its organic salt-ibuprofenate amino acid alkyl ester on its biodegradation.

The stimulus to take up this topic was the lack of information on the biodegradation of ionic liquids. As was shown previously, the obtained compounds, due to their chemical structure and melting point below 100 °C, belong to the group of ionic liquids. The conversion of solid active pharmaceutical ingredients into liquid forms is very promising. The literature mentions a special group of ionic liquids called ionic liquid comprising active pharmaceutical ingredients (API-ILs). API-ILs can be used, among others, in synthesis (as a solvent, co-solvent, reagent, catalyst, or enantioselectivity enhancers), crystallization, solvents, co-solvents, or emulsifiers for API solubilization. Moreover, ionic liquids may display specific biological activities, therefore they are potential pharmaceutical substances [24,25,26]. Ionic liquids can also be used in drug delivery systems [27,28].

Ionic liquids used as active substances contained in pharmaceutical formulae should be characterized by low toxicity and high biodegradability. According to biodegradation tests recommended by the OECD, only paracetamol and salicylic acid derivatives are considered biodegradable by active sludge. Due to the low biodegradability of nonsteroidal anti-inflammatory drugs, the research has been conducted for several years on the intensification of degradation processes involving active sludge [29].

The literature on the subject states that ionic liquids containing short, side alkyl chains in their structure are characterized by low toxicity and high resistance to biodegradation [30]. Other reports in the literature indicate that ionic liquids with substituents from one to five carbon atoms are relatively less toxic than liquids with substituents from seven and more carbon atoms. This relationship also applies to biodegradability as ionic liquids are more biodegradable with short-chain alkyl substituents [31,32]. However, apart from the length of the side alkyl chains, the susceptibility of ionic liquids to biodegradation can be increased by the appropriate choice of substituents present in the structure of ionic liquids [33,34]. Ester groups greatly increase the susceptibility of the compound to degradation, while amide groups reduce the susceptibility of the test compound to biodegradation [34,35]. The challenge, therefore, is to combine the low toxicity of the resulting compound with its high biodegradability. Biodegradation of active pharmaceutical amino acid alkyl ester derivatives has not been described so far.

**Figure 1:**
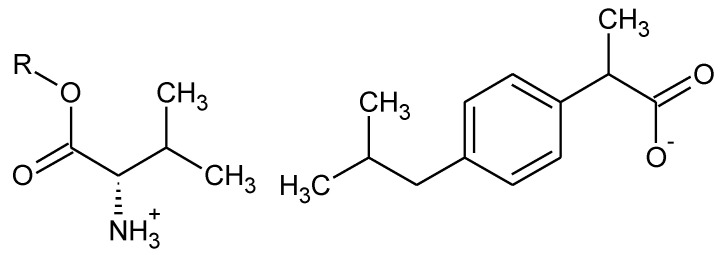
The structures of ibuprofen salts with L-valine alkyl esters, R = C_1_–C_8_.

The present study was carried out to determine the effect of the structure of the organic cation (Figure 1), in particular, the alkyl chain length, on the biodegradation process of these compounds. In this study, properties such as water solubility and partition coefficient were correlated with the biodegradability results.

## 2. Materials and Methods

### 2.1. Ionic Liquids Used

All ionic liquids used in the research were obtained in accordance with the methodology described in our previous publication [22,23]. The synthesis consisted of three steps. In the first step, the esterification and the chloro-hydrogenation reactions of amino acids with the use of alcohol and a chlorinating agent were run simultaneously, respectively. The obtained amino acid alkyl ester hydrochloride was then neutralized with a 25% ammonia solution. In the last step, the obtained ester was reacted with ibuprofen. The reactions were carried out in diethyl ether. All obtained compounds were fully characterized and described. Identity studies were confirmed based on the analysis of ^1^H NMR, ^13^C NMR, FTIR, UV–Vis spectra, and all the necessary spectroscopic data have been described previously [22,23].

### 2.2. Elemental Analysis

The elemental analysis CHNS/O was performed by using a Thermo Scientific™ FLASH 2000 CHNS/O Analyzer (Waltham, MA, USA). Compounds were weighed to an accuracy of ±0.000001 g in tin crucibles (2–3 mg) for analysis in CHNS mode, and in silver crucibles (1–2 mg) in oxygen mode, respectively. 2,5-(Bis(5-tert-butyl-2-benzo-oxazol-2-yl) thiophene (BBOT), sulfanilamide, L-cysteine, and L-methionine were used as standards to calibrate the device in CHNS mode. In oxygen mode, acetanilide and benzoic acid were used.

### 2.3. Chemicals and the Test Medium

For the determination of biodegradability and to assess the lipophilicity of L-valine alkyl ester ibuprofenates, KH_2_PO_4_, K_2_HPO_4_, Na_2_HPO_4_·2H_2_O, NH_4_Cl, MgSO_4_·7H_2_O, CaCl_2_·2H_2_O, KOH, NaOH, HCl, and FeCl_3_·6H_2_O were purchased from Chempur, Piekary Śląskie (Poland). All other chemicals were of the highest purity commercially available.

The test medium was prepared from the following solutions (per liter): 10 mL of solution a—adjusted to pH 7.4 (per liter): 8.5 g of KH_2_PO_4_, 21.75 g of K_2_HPO_4_, 33.4 g of Na_2_HPO_4_·2H_2_O, and 0.5 g of NH_4_Cl; 1 mL of solution b (per liter): 22.5 g of MgSO_4_·7H_2_O; 1 mL of solution c (per liter): 36.4 g of CaCl_2_·2H_2_O; 1 mL of solution d (per liter): 0.25 g of FeCl_3_·6H_2_O.

### 2.4. Origin of Active Sludge Samples

**Figure 2:**
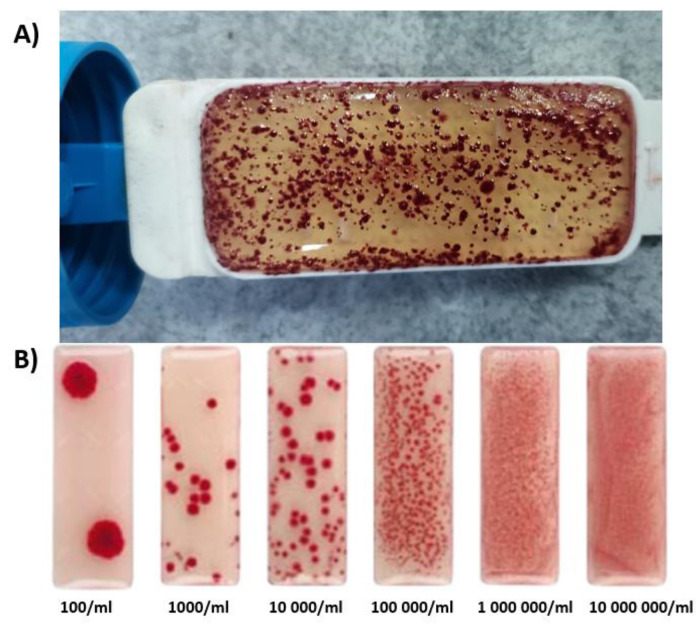
(**A**) Appearance of the test obtained after immersion of the insert in the active sludge (the number of bacteria per 1 mL of active sludge after a 96-h observation). (**B**) Appearance of the reference test.

Active sludge samples were collected from the sewage treatment plant “Pomorzany” from the aeration chamber; later aerated, and stored until use. The concentration of active sludge suspensions was subjected to a microbiological test used for the designation of the total number of microorganisms (Schulke Mikrocount Duo, Norderstedt, Germany). A microbiological test with medium and TTC agar was immersed for 10 s in an active sludge. The test was secured at room temperature for four days, after which the number of bacteria was evaluated (by evaluating the appearance of the test against the appearance of a benchmark test)—Figure 2.

The organic compounds (IBU, [ValOMe][IBU], [ValOEt][IBU], [ValOPr][IBU], [ValOiPr][IBU], [ValOBu][IBU], [ValOAm][IBU], [ValOHex][IBU], [ValOHept][IBU], [ValOOct][IBU], SDS) were the only sources of carbon and energy (concentration—40 mg/L organic carbon) and were tested in duplicate.

Biodegradation in the mineral medium by CO_2_ production—General method for determining aerobic biodegradation potential [29,36,37,38,39].

**Figure 3:**
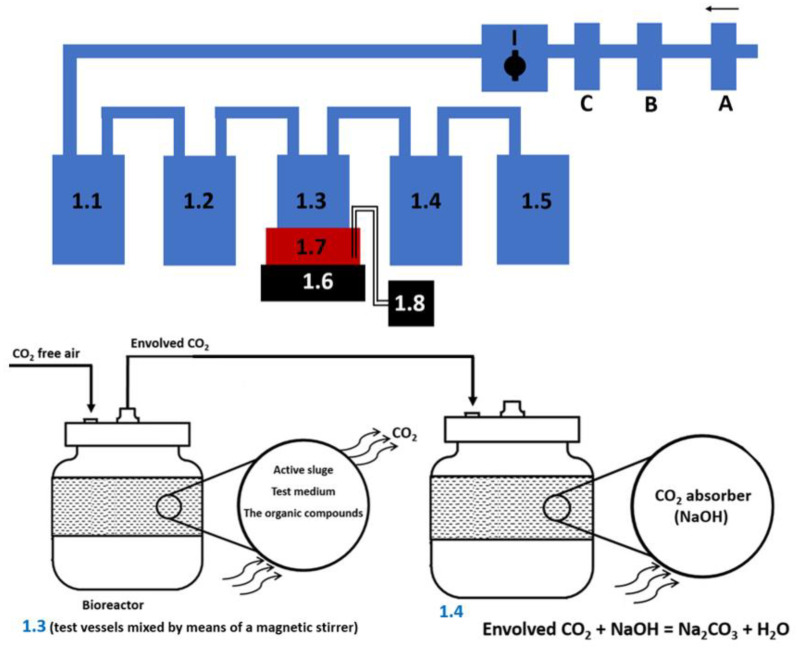
The test system to measure carbon dioxide.

First, the test vessels were set up in line (1.1, 1.2, 1.3, 1.4, 1.5), as shown in Figure 3, and a magnetic stirrer was also installed (1.6). Then, the vessels were connected with tubes. Compressed air (A) flowing through CO_2_ absorbers (B, C) aerated the test system. Air, whose speed was adjusted by a valve (I) was directed to a CO_2_ absorber (1.1), then to a CO_2_ indicator (1.2), aiming to indicate any CO_2_ in the air by turbidity.

The test system to measure carbon dioxide is shown in Figure 3.

In the test vessel, 1.3, we placed: 250 mL of the test medium, 2.5 mL of active sludge, and organic compound in a quantity corresponding to 40 mg/L of organic carbon. The concentrations of the starting compounds IBU, [ValOMe][IBU], [ValOEt][IBU], [ValOPr][IBU], [ValOiPr][IBU], [ValOBu][IBU], [ValOAm][IBU], [ValOHex][IBU], [ValOHept][IBU], [ValOOct][IBU], SDS, were respectively: 53.24, 59.16, 58.16, 57.96, 57.96, 57.44, 57.00, 56.56, 56.16, 55.80, and 84.12 mg/L.

As a result of the biodegradability of the compound, the vessel 1.3 produced carbon dioxide, which reacts with NaOH (1.4), to produce Na_2_CO_3_:

NaOH + CO_2_ = Na_2_CO_3_ + H_2_O, (1)

To determine the amount of carbon dioxide in the vessel (1.3), 10 mL solution from 1.4 was collected in a 25 mL flask. The contents of the flask were replenished with deionized water to the established limit. The sample was then analyzed using a total organic carbon analyzer TOC-LCSH/CSN, Shimadzu Corporation:

Na_2_CO_3_ + 2 HCl = CO_2_ + 2 NaCl + H_2_O, NaHCO_3_ + HCl = CO_2_ + NaCl + H_2_O, (2)

First, the test sample contains carbonates and acidic carbonates acidified with hydrochloric acid (to obtain pH < 3). Carbonates and acidic carbonates are converted into carbon dioxide. Thus, the amount of inorganic carbon in the test sample was obtained. The test system to measure carbon dioxide is shown in Figure 3 and it consisted of the following elements: A: compressed air, aeration of the test system (aeration rate from 50 to 100 mL/min), B, C: CO_2_ absorber (KOH), I: aeration rate control valve a test system, 1.1: CO_2_ absorber (KOH)—concentration 10 mol/L, 1.2: CO_2_ indication (Ba(OH)_2_)—concentration 0.01 mol/L, 1.3: test vessels with a capacity of 500 mL mixed through a magnetic stirrer 1.6, 1.4: CO_2_ absorber (NaOH)—concentration 0.05 mol/L, 1.5: O_2_ absorber (H_2_O), 1.7: plastic container with cryostat 1.8. Test vessel with a capacity of 500 mL placed in a plastic container. The vessels were equipped with a cryostat in order to accurately determine the temperature of the water bath. Incubation was carried out at a temperature of 23 °C. Test results obtained are shown in Table 1 and Table 2.

### 2.5. HPLC Analysis

The content of the test compound in the test medium (test vessel 1.3, Figure 3), solubility determinations, and concentration in the water phase in the partition coefficient experiments were determined using a Shimadzu, model Nexera-i, LC-2040C 3D PLUS HPLC system (Kyoto, Japan), equipped with a UV–VIS/DAD detector and Kinetex^®^F5 column (2.6 μm; 150 × 4.6 mm; Phenomenex, Torrance, CA, USA).

Analyses were performed at 35 °C under isocratic conditions, with the mobile phase consisting of the water–acetonitrile mixture (50/50, *v*/*v*) and a flow rate of 1 cm^3^/min. The detection wavelength was 210 nm. Data acquisition and processing were performed using a LabSolutions/LC Solution System (LabSolutions Lite, 5.93, Shimadzu (Kyoto, Japan)). Injections were repeated at least three times for each sample and the results were averaged. The concentration of ibuprofen and its salts was calculated on peak area measurements using a calibration curve method.

### 2.6. Solubility Experiments

The solubility of ibuprofen and L-valine alkyl ester ibuprofenates in deionized water and phosphate buffer (7.4—corresponding to the concentration in test vessel 1.3, Figure 3) were determined. An excess of substance was added to 2 cm^3^ of water or buffer in a screwed vial and was stirred vigorously at 25.00 ± 0.05 °C or 32.00 ± 0.05 °C for 24 h. Then, the mixture was centrifuged at the respective temperature, and liquid above the solid was taken, diluted, and analyzed by the HPLC method to determine the concentration of the substance.

### 2.7. Determination of Partition Coefficient

To determine the partition coefficient for ibuprofen and L-valine alkyl ester ibuprofenates, 10 mg (with an accuracy of 0.01 mg) of the respective compound was weighed. Then, 5 cm^3^ water (or buffer) saturated with n-octanol and 5 cm^3^ of n-octanol saturated with water were added. The mixture was vigorously agitated at 25 °C for 3 h followed by centrifugation at 7500 rpm, at 25 °C for 10 min for better phase separation. After centrifugation, the aqueous layer was decanted and analyzed by HPLC to determine the concentration of the compound. The partition coefficient *log P_ow_* was calculated following the formula:

*log P_ow_* = *log c_oct_* − *log c_w_*, (3)

where *c_w_* and *c_oct_* represent concentration (mg/dm^3^) of the substance dissolved in the aqueous layer (water or phosphate buffer) and octanol, respectively.

The concentration of the compound dissolved in octanol was calculated by the formula:

*C_oct_* = *c*_0_ − *c_w_* (mg/dm^3^), (4)

where *c*_0_ is a total concentration (mg/dm^3^), calculated based on the mass of compound used in the experiment.

## 3. Results and Discussion

### 3.1. Elemental Analysis

Before starting the biodegradation tests, the organic carbon content was confirmed by elemental analysis. The results of the content of carbon, hydrogen, nitrogen, sulfur, and oxygen in the tested compounds are presented below.

Elemental analysis (%) for IBU: C 75.69, H 8.80, N 0.00, O 15.51, for [ValOMe][IBU]: C (67.63), H (9.26), N (4.11), O (18.18), for [ValOEt][IBU]: C 68.25, H 9.47, N 3.92, O 18.18, for [ValOPr][IBU]: C 68.80, H 9.70, N 3.82, O 17.50, for [ValOiPr][IBU]: C 69.23, H 9.65, N 3.86, O 17.45, for [ValOBu][IBU]: C 69.64, H 9.82, N 3.67, O 16.77, for [ValOAm][IBU]: C 70.08, H 9.99, N 3.74, O 16.25, for [ValOHex][IBU]: C 70.39, H 10.10, N 3.44, O 15.64, for [ValOHept][IBU]: C (71.22), H (10.30), N (3.04), O (15.14), for [ValOOct][IBU]: C (71.64), H (10.43), N (3.08), O (14.63), for SDS: C 46.14, H 98.13, N 0.00, O 24.58, S 12.32.

The obtained results confirm that only pure compounds were used in the research.

### 3.2. Biodegradation Studies

The number of bacteria (per 1 mL of active sludge) was about 100,000 (after a 96-h observation)—Figure 2.

**Figure 4:**
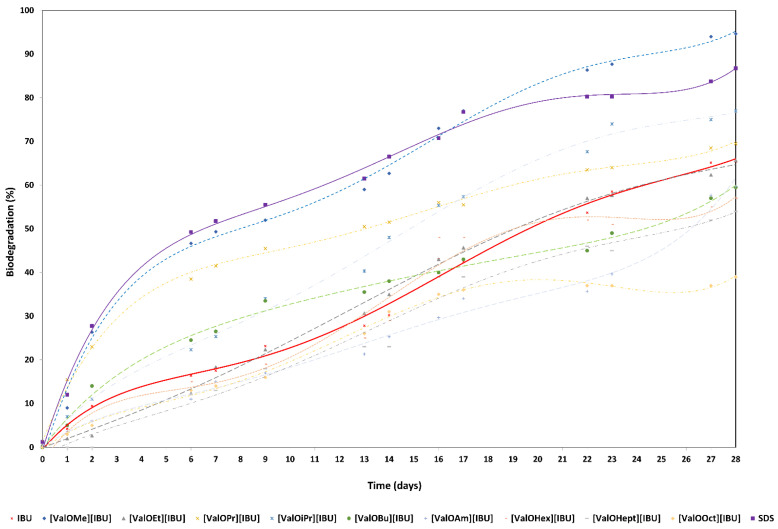
Biodegradation curves for the ibuprofen (red line), L-valine alkyl ester ibuprofenates, and SDS (as the reference compound—purple line).

Degradation of ibuprofen, L-valine alkyl ester ibuprofenates, and SDS (Figure 4).

Tables S1 and S2 in the Supplementary Materials present the biodegradation of ionic liquids based on ibuprofen and SDS (as a reference compound) by bacterial cultures.

The maximum level of biodegradation after 28 days was 65.4% ± 3.1 of ibuprofen. The highest degree of biodegradability (94.7% ± 2.3) was assigned to [ValOMe][IBU], and slightly lower degrees of biodegradation (86.8% ± 8.5) were characterized by the reference compound—sodium dodecyl sulfate (SDS). Furthermore, five modified compounds [ValOEt][IBU], [ValOPr][IBU], [ValOiPr][IBU], [ValOBu][IBU], and [ValOAm][IBU] were characterized readily by biodegradabilities of 65.7% ± 0.6, 69.5% ± 10.6, 77.0% ± 1.0, 59.5% ± 9.8, and 59.7% ± 10.6, respectively. While extending the side alkyl chain, poorly biodegradation susceptibility of tested compounds was observed: [ValOHex][IBU] (57.0% ± 3.4), [ValOHept][IBU] (54.0% ± 1.5), and [ValOOct][IBU] (39.0% ± 5.4) after 28 days of the experiment (Figure 4, Tables S1 and S2).

Half-life of ibuprofen and L-valine alkyl esters ibuprofenate by bacterial cultures (Table 1).

Table 2 presents the phase of degradation of ionic liquids based on ibuprofen and SDS.

The half-life of ibuprofen was 20.5 days (Table S1), whereas after 33 h of conducting tests involving IBU, a 10% degradation of the test compound was achieved (Table 2). It is therefore classified as easily degradable. Furthermore, four modified compounds [Va-lOMe][IBU], [ValOEt][IBU], [ValOPr][IBU], [ValOiPr][IBU] occurring as ionic liquids and SDS were characterized by a shorter half-life of 12.7, 19.3, 12.9, 14.5, and 6.8 days, respectively, than standard ibuprofen (Table 1). The compounds containing 4, 5, 6, 7, and 8 carbon atoms within the side chain [ValOBuIBU], [ValOAmIBU], [ValOHex][IBU], [ValOHept][IBU], and [ValOOct][IBU] were characterized by a longer half-life than the original ibuprofen. The half-life of these compounds was 23.7, 23.4, 22.5, 26.0, and 35.9 days, respectively (Table S1).

The compound containing the propyl group [ValOPr][IBU] in its structure and sodium dodecyl sulfate reached 10% degradation already after 15 h, however, [Va-lOMe][IBU] containing the shortest side alkyl chain in its structure reached 10% degradation after 17 h. In contrast, the [ValOiPr][IBU] containing the isopropyl group reached 10% degradation not until 34 h later (due to adaptations of the lag phase microorganisms). Longer adaptation times for microorganisms in the lag phase are required by the following compounds: [ValOEt][IBU] (118 h), [ValOAm][IBU] (48 h), [ValOHex][IBU] (46 h), [ValOHept][IBU] (65 h), [ValOOct][IBU] (37 h); see Table S2.

Degradation of the ibuprofen and its esters by active sludge was indicated by the consumption of the tested compounds after 68 days of conducting the process.

**Figure 5:**
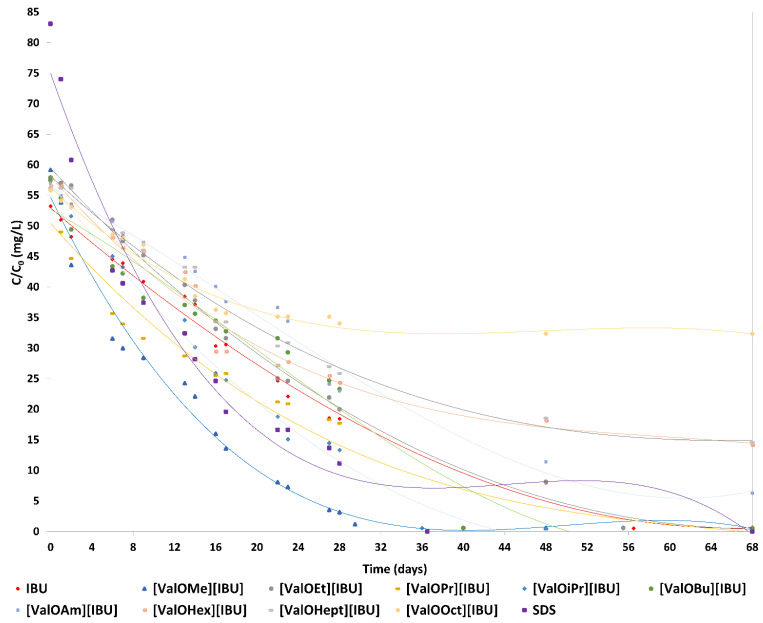
Degradation profiles of ibuprofen, the alkyl esters of L-valine, and SDS by bacterial cultures. Experimental conditions: IBU= 53.24 mg/L, [ValOMe][IBU] = 59.16 mg/L, [ValOEt][IBU] = 58.16 mg/L, [ValOPr][IBU] = 58.00 mg/L, [ValOiPr][IBU] = 57.96 mg/L, [ValOBu][IBU] = 57.44 mg/L, [ValOAm][IBU] = 57.00 mg/L, [ValOHex][IBU] = 56.56 mg/L, [ValOHept][IBU] = 56.16 mg/L, [ValOOct][IBU] = 55.80 mg/L, SDS = 84.12 mg/L.

As shown in Figure 5, bacterial cultures are capable of degrading ibuprofen, L-valine alkyl ester ibuprofenates, and SDS. Almost 100% of IBU (52.71 mg/L), [ValOMe][IBU] (58.57 mg/L), [ValOEt][IBU] (57.58 mg/L), [ValOPr][IBU] (57.38 mg/L), [ValOiPr][IBU] (57.38 mg/L), [ValOBu][IBU] (56.87 mg/L), and SDS (84.11 mg/L) was degraded within 68 days.

A total of 89.0% [ValOAm][IBU], 75.0% [ValOHex][IBU], and 74.0% [ValOHept][IBU] degradation was observed after 68 days, in contrast, only 42.0% [ValOOct][IBU] degradation was observed after 68 days. These results indicate the bacterial cultures are active for both ibuprofen, the alkyl esters of L-valine, and SDS.

Between 1–28 days of conducting the degradation rate of the tested compounds was the highest and was respectively: 2.3% for IBU and for [ValOEt][IBU], 3.4% for [ValOMe][IBU], 2.5% for [ValOPr][IBU], 2.8% for [ValOiPr][IBU], 2.1% [ValOBu][IBU] and for [ValOAm][IBU], 2.0% for [ValOHex][IBU], 1.9% for [ValOHept][IBU], 1.4% [ValOOct][IBU], and 3.1% for the reference compound.

At 29–68 days, the degradation rate of [ValOBu][IBU], [ValOAm][IBU], [ValOHex][IBU], [ValOHept][IBU] and [ValOOct][IBU] by active sludge decreased (amounted to ≤1%/day). In the case of IBU, [ValOMe][IBU], [ValOEt][IBU], [ValOPr][IBU], and [ValOiPr][IBU], the degradation rate by active sludge amounted to ≥1%/day.

### 3.3. Solubility Experiments

Dependence of half-life of the alkyl esters of L-valine on its solubility.

**Figure 6:**
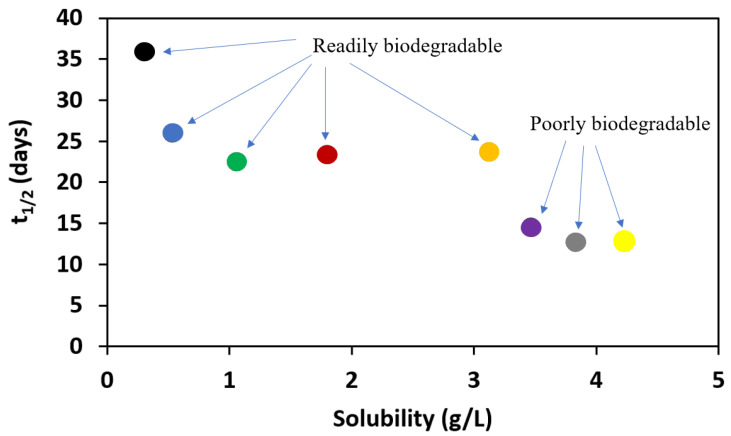
Dependence of half-life of the alkyl esters of L-valine on its solubility: [ValOMe][IBU]—black dot, [ValOEt][IBU]—blue dot, [ValOPr][IBU]—green dot, [ValOBu][IBU]—red dot, [ValOAm][IBU]—orange dot, [ValOHex][IBU]—violet dot, [ValOHept][IBU]—grey dot, [ValOOct][IBU]—yellow dot.

It can be further noted that half-lives for these compounds depend on the solubility of these compounds. In biodegradation studies, poor solubility may result in low bioavailability and thus lower biodegradation rates, as shown in Figure 6.

### 3.4. Determination of Partition Coefficient

**Figure 7:**
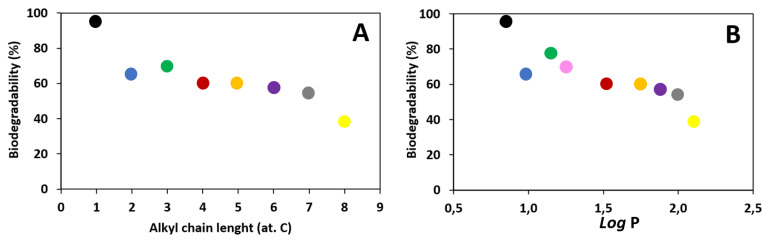
Dependence of biodegradability (**A**) on the alkyl chain length, (**B**) on the lipophilicity (expressed as *log* P): [ValOMe][IBU]—black dot, [ValOEt][IBU]—blue dot, [ValOPr][IBU]—green dot, [ValOiPr][IBU]—pink dot, [ValOBu][IBU]—red dot, [ValOAm][IBU]—orange dot, [ValOHex][IBU]—violet dot, [ValOHept][IBU]—grey dot, [ValOOct][IBU]—yellow dot.

Dependence of biodegradability on the alkyl chain length and the lipophilicity in the alkyl esters of L-valine (Figure 7).

**Figure 8:**
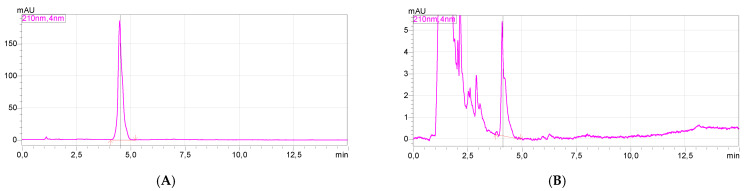

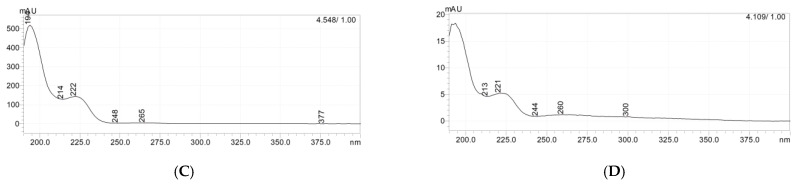
HPLC chromatograms (**A**,**B**) and UV–Vis spectra (**C**,**D**) of [ValOAm][IBU] (reference, at (**A**,**C**)) and in the test ves-sel after 28 days of biodegradation at (**B**,**D**).

It has been shown that the length of the alkyl chain in the cationic part of the ionic liquid (in the alkyl ester of L-valine) affects biodegradability. A significant decrease in biodegradability was noticed with an increase in the length of the carbon chain, as shown in Figure 8A. Moreover, a relationship was observed between the lipophilicity of the analyzed compounds, expressed as the partition coefficient (*log* P) and biodegradability (Figure 8B). The obtained results indicate that the less lipophilic the compound, the higher the biodegradation. The reason for this phenomenon is probably the higher toxicity against bacteria from activated sludge of these compounds [40,41].

### 3.5. HPLC Analysis

HPLC analysis of the solutions in the test vessel after 28 days of biodegradation showed that the test compound could still be detected in the test vessel after the test time. For confirmation, the chromatogram and the UV–Vis spectrum of the test compound are shown (Figure 8, at left). The right side of Figure 6 shows the chromatogram and the UV–Vis spectrum for the signal with a retention time of 4.109 min; the remaining peaks visible in this chromatogram come from the test mixture and are also detected in the blank. As can be seen, the obtained UV–Vis spectra are identical, therefore the analyzed compound was [ValOAm][IBU]. A similar situation was observed for the remaining compounds.

The presence of intermediates (hydroxylated derivatives) in the test vessel may be responsible for the decreased degradation rate of ibuprofen and its esters [29,36,37,38,39]. The major metabolites of ibuprofen are 1-hydroxyibuprofen, 2-hydroxyibuprofen, and 1,2-dihydroxyibuprofen. There are not many literature reports on the degradation of non-steroidal anti-inflammatory drugs in aqueous media. Mostly, only the initial stages of biotransformation of these compounds are known [29,41,42,43].

In contrast, biodegradability tests of enantiomers of ibuprofen carried out in accordance with OECD and using active sludge showed that the degradation of (*R*,*S*)-ibuprofen started only after five days of incubation. In addition, the results showed that the enantiomers of ibuprofen were recognized by the microorganisms present in the sludge because the biodegradation of (*R*)-ibuprofen was higher than for (*S*)-ibuprofen. The maximum level of biodegradation after 28 days of the experiment and the half-lives for these compounds were 68% in 18 days and 50% in 25 days, respectively [44]. In contrast, mineralization of ibuprofen performed in accordance with OECD due to adaptations of the lag phase microorganisms was less than 10% on the sixth day of experimentation. Ibuprofen thus did not inhibit the microbial activity at the applied concentration. Results clearly show that ibuprofen is readily biodegradable in aqueous systems, and, based on these data, apparently does not pose a risk for the environment [9,45,46,47,48,49]. Other reports in the literature also indicate that when carrying out anaerobic degradation of ibuprofen for 112 days and at a temperature of 37 °C (by methanogenic bacteria), there was no significant degradation of the test compound [50].

The research conducted by Coleman et al. [34] showed that the values of the degree of biodegradability of SDS were also high at about 95% after 28 days of the experiment. In the case of the compounds containing the propyl group [PrOCH_2_CH_2_OCOCH_2_min][OctOSO_3_] and butyl group [BuOCH_2_CH_2_OCOCH_2_min][OctOSO_3_], the degree of biodegradability (determined by CO_2_ released and during 0 to 28 days) was more than 60% [34]. The ionic liquid [EtOCH_2_CH_2_OCOCH_2_min][OctOSO_3_] containing the ethyl group had a slightly lower biodegradability value of 59% [34].

Functionalizing of long alkyl chains could be susceptibility to the degradation of ionic liquids. The introduction of ether, ester, ester, and ether groups (simultaneously), into long had alkyl chains improved the ecotoxicity of the ionic liquids as well as nitrile, hydroxyl, and ether groups on the alkyl chain and the double bond [34,51,52,53,54,55].

In 2014, Steudte et al. reported that the use of dicationic ionic liquids with higher hydrophilic moieties (thus replacing monovalent ions) are still less toxic than the monovalent cationic ILs [56,57].

## 4. Conclusions

In this study, microorganism activity was used to assess the ibuprofen and L-valine alkyl ester ibuprofenates’ biodegradability (under aerobic conditions) to determine whether they are persistent in the environment. A method to measure the biodegradability of these compounds, recommended by the OECD, was proposed as well as the aerobic biodegradability of 11 organic compounds, and assessed by measuring the CO_2_ released. The degree of biodegradability was calculated. The results were presented as the average value (with standard deviations) obtained from three series of tests. According to the degree of biodegradability value, ibuprofen and L-valine alkyl ester ibuprofenates were classified as readily and poorly biodegradable. As expected, a correlation between increasing chain length and increasing lipophilicity was found. It has also been shown that biodegradation decreases with increasing lipophilicity.

Our research on the assessment of the biodegradation of ibuprofen and L-valine alkyl ester ibuprofenates by bacterial cultures showed that eight of the 11 compounds tested (IBU, [ValOMe][IBU], [ValOEt][IBU], [ValOPr][IBU], [ValOiPr][IBU], [ValOBu][IBU], [ValOAm][IBU], and SDS) constitute an attractive source of carbon and energy for the microorganisms used and were easily biodegradable.

The biodegradability of these new, potentially very interesting, alternative compounds to the currently used ibuprofen has been shown to be dependent on the length of the ester chain of the amino acid cation. The most biodegradable compounds had a short alkyl chain. Considering our previous results, the permeability of these compounds through the skin, and the biodegradability results obtained now, the most promising modification is the use of isopropyl and propyl esters of amino acids.

The presented work is the basis for future research on the influence of the structure of amino acid ionic liquids including the type of amino acid used and the influence of alkyl chain branching on the biodegradation process.

## Figures and Tables

**Table 1:** Half-life of ibuprofen and L-valine alkyl esters ibuprofenate by bacterial cultures.

The Half-Life of the Analyzed Compound					
IBU	[ValOMe][IBU]	[ValOEt][IBU]	[ValOPr][IBU]	[ValOiPr][IBU]	[ValOBu][IBU]
491.6 h	305.8 h	463.2 h	308.9 h	347.0 h	568.4 h
20.5 days	12.7 days	19.3 days	12.9 days	14.5 days	23.7 days
The Half-Life of the Analyzed Compound					
[ValOAm][IBU]	[ValOHex][IBU]	[ValOHept][IBU]	[ValOOct][IBU]	SDS	
(h/days)					
561.8 h	541.2 h	623.1 h	861.5 h	162.3 h	
23.4 days	22.5 days	26.0 days	35.9 days	6.8 days	

**Table 2:** Phase of degradation of ibuprofen and L-valine alkyl ester ibuprofenates by bacterial cultures.

Compound Name	Phase of Degradation (%/h)		
	Lag Phase	Degradation Phase	Plateau Phase
IBU	0–7	7–59	59–65
	0–33	33–556	556–672
[ValOMe][IBU]	0–9	9–85	85–95
	0–17	17–536	536–672
[ValOEt][IBU]	0–7	7–59	59–65
	0–118	118–566	566–672
[ValOPr][IBU]	0–7	7–63	63–70
	0–15	15–539	539–672
[ValOiPr][IBU]	0–8	8–69	69–77
	0–34	34–517	517–672
[ValOBu][IBU]	0–6	6–54	54–60
	0–20	20–603	603–672
[ValOAm][IBU]	0–6	6–54	54–60
	0–48	48–603	603–672
[ValOHex][IBU]	0–6	6–51	51–57
	0–46	46–555	555–672
[ValOHept][IBU]	0–5	5–49	49–54
	0–65	65–596	596–672
[ValOOct][IBU]	0–4	4–35	35–39
	0–37	37–524	524–672
SDS	0–9	9–78	78–87
	0–15	15–537	537–672

SDS—sodium dodecyl sulfate (reference compound).

## Data Availability

Any data related to the study can be provided on reasonable request.

## Supplementary Materials

The following are available online at https://www.mdpi.com/article/10.3390/ma14123180/s1, Table S1: Biodegradation of ibuprofen and L-valine alkyl ester ibuprofenates by bacterial cultures, Table S2: Biodegradation of ibuprofen and L-valine alkyl ester ibuprofenates by bacterial cultures.
