# Supplementary material for: Biodegradation of L-Valine Alkyl Ester Ibuprofenates by Bacterial Cultures
Source: Materials (Basel). 2021 Jun 9;14(12):3180. doi: 10.3390/ma14123180 (PMC8228323; doi:10.3390/ma14123180)
Supplement: Supplementary file 1 [file materials-14-03180-s001.zip › materials-1234159-supplementary.pdf]

materials

MDPI

Supplementary Materials

Biodegradation of L-valine alkyl ester ibuprofenates by bacterial cultures

Edyta Makuch [1], Paula Ossowicz-Rupniewska [1,*], Joanna Klebeko [1] and Ewa Janus [1]

[1] Faculty of Chemical Technology and Engineering, Department of Chemical Organic Technology and Polymeric Materials, West Pomeranian University of Technology, Szczecin, PL-70322 Szczecin, Poland; possowicz@zut.edu.pl; emakuch@zut.edu.pl; joanna.klebeko@gmail.com; ejanus@zut.edu.pl

* Correspondence: possowicz@zut.edu.pl; Tel.: +48-91-449-48-01

Table S1. Biodegradation of ibuprofen and L-valine alkyl ester ibuprofenates by bacterial cultures.

Time (days)	*Biodegradation after 3 cycles of tests					
	IBU	[ValOMe][IBU] 1**	[ValOEt][IBU] 2**	[ValOPr][IBU] 3**	[ValOiPr][IBU] 3**	[ValOBu][IBU] 4**
	(%)					
0	0 ± 0.00	0 ± 0.00	1 ± 1.15	0 ± 0.00	0 ± 0.00	0 ± 0.00
1	4 ± 2.32	9 ± 11.53	2 ± 1.73	16 ± 4.95	7 ± 2.00	5 ± 0.76
2	9 ± 3.71	26 ± 11.68	3 ± 1.53	23 ± 0.00	11 ± 1.00	14 ± 2.60
6	16 ± 9.81	47 ± 5.51	12 ± 6.51	39 ± 10.61	22 ± 5.51	25 ± 4.68
7	18 ± 10.02	49 ± 5.86	18 ± 3.51	42 ± 10.61	25 ± 6.51	27 ± 4.46
9	23 ± 8.48	52 ± 5.29	22 ± 0.58	46 ± 12.02	34 ± 4.58	34 ± 4.85
13	28 ± 6.66	59 ± 5.29	31 ± 1.15	51 ± 13.44	40 ± 7.57	36 ± 5.20
14	30 ± 7.38	63 ± 3.51	35 ± 1.73	52 ± 12.02	48 ± 6.08	38 ± 6.02
16	43 ± 7.57	73 ± 1.73	43 ± 1.73	56 ± 11.31	55 ± 5.03	40 ± 6.59
17	43 ± 11.06	77 ± 1.00	46 ± 4.04	56 ± 12.02	57 ± 2.31	43 ± 7.07
22	54 ± 7.54	86 ± 3.06	57 ± 6.93	64 ± 4.95	68 ± 9.29	45 ± 7.94
23	58 ± 6.73	88 ± 1.53	58 ± 5.77	64 ± 4.24	74 ± 3.46	49 ± 8.10
27	65 ± 2.48	94 ± 2.65	62 ± 1.15	69 ± 12.02	75 ± 2.65	57 ± 9.48
28	65 ± 3.12	95 ± 2.31	66 ± 0.58	70 ± 10.61	77 ± 1.00	60 ± 9.76
Qualitative assessment of aerobic biodegradability	Readily biodegradable	Readily biodegradable	Readily biodegradable	Readily biodegradable	Readily biodegradable	Readily biodegradable

* Mean ± SD (n = 6), ** length of alkyl chain in ester part.

Table S2. Biodegradation of ibuprofen and L-valine alkyl ester ibuprofenates by bacterial cultures.

**Time (days)**	***Biodegradation after 3 cycles of tests**				
	**[ValOAm][IBU] 5****	**[ValOHex][IBU] 6****	**[ValOHept][IBU] 7****	**[ValOOct][IBU] 8****	**SDS**
	**(%)**				
0	0 ± 0.00	0 ± 0.00	0 ± 0.00	0 ± 3.60	1 ± 1.50
1	4 ± 2.12	0 ± 0.02	0 ± 0.36	3 ± 1.92	12 ± 8.91
2	6 ± 4.24	6 ± 0.95	0 ± 2.27	5 ± 1.77	28 ± 10.24
6	11 ± 9.19	15 ± 2.13	12 ± 1.89	13 ± 1.23	49 ± 2.87
7	15 ± 7.78	18 ± 1.98	13 ± 1.80	14 ± 1.27	52 ± 5.06
9	17 ± 10.61	19 ± 2.82	18 ± 0.73	16 ± 5.43	56 ± 3.70
13	21 ± 17.68	25 ± 5.59	23 ± 1.85	26 ± 3.83	62 ± 3.11
14	25 ± 12.73	29 ± 7.67	23 ± 1.68	31 ± 2.70	67 ± 2.89
16	30 ± 12.02	48 ± 9.36	39 ± 3.72	35 ± 3.31	71 ± 2.22
17	34 ± 14.14	48 ± 11.03	39 ± 2.63	36 ± 5.28	77 ± 4.19
22	36 ± 10.61	52 ± 3.88	46 ± 2.14	37 ± 5.07	80 ± 6.29
23	40 ± 7.07	51 ± 4.92	45 ± 0.75	37 ± 6.74	80 ± 6.50
27	58 ± 7.78	55 ± 2.97	52 ± 1.06	37 ± 6.65	84 ± 8.85
28	60 ± 10.61	57 ± 3.39	54 ± 1.49	39 ± 5.43	87 ± 8.54
Qualitative assessment of aerobic biodegradability	Readily biodegradable	Poorly biodegradable	Poorly biodegradable	Poorly biodegradable	Readily biodegradable

SDS - sodium dodecyl sulfate (reference compound),
* Mean ± SD (n = 6), ** length of alkyl chain in ester part.
